# Going hard and early: Aotearoa New Zealand's response to Covid-19

**DOI:** 10.1017/S174413312100013X

**Published:** 2021-03-05

**Authors:** Jacqueline Cumming

**Affiliations:** Health Services Research and Policy Consultant

**Keywords:** COVID-19, Health Policy – COVID-19, New Zealand

## Abstract

Aotearoa New Zealand went ‘hard’ and ‘early’ in its response to COVID-19 and has been highly successful in limiting the spread and impact of the virus. The response has ramped up over time, and has included various levels of: border control; advice on hygiene, physical distancing and mask wearing; advice to remain at home if unwell; and testing and tracing. A four-level Alert Level framework has guided key actions at different levels of risk. Strong leadership from the Prime Minister, Minister of Finance, and Director-General of Health and high levels of community co-operation have supported the response. The country is most vulnerable at its borders, where arrangements have been of concern; advice on testing and the wearing of masks has changed over time; while the use and distribution of personal protective equipment has also been of concern. The country overall was not well prepared for a pandemic, but policy-making has been nimble. Key challenges for 2021 include swiftly rolling out a vaccine, catching up on delayed health care, and deciding how and when the border can reopen. The economic, and associated social, challenges will last many years.

## 1. Introduction

Aotearoa New Zealand's (A/NZ's) response to COVID-19 has been described by the government as going ‘hard’ and ‘early’, with the goal of eliminating transmission of virus in the community, and in ways that ensure equity (Ministry of Health, 2020*a*, 2020*b*). The country has been highly successful at limiting the spread and impact of the virus. By 18 January 2021, there had been a total of 1906 confirmed and 356 probable cases (a total of 2262 cases), with 25 deaths (Ministry of Health, 2021) for a population of 5.1 million. (Note that ‘Aotearoa’ in an Indigenous name for New Zealand.)

In this paper, I provide information on the decision-making processes involved with COVID-19 in A/NZ (Section 2); an overview and timeline of A/NZ's approach to and experiences with COVID-19 (Section 3); discuss the range of measures that the A/NZ government has taken to combat the virus and the issues faced, focusing on International Borders; Restrictions; Cases, Clusters and Deaths; Tracing and Testing; and the Capacity of the Health System (Sections 4–8); explore the economic impact of COVID-19 and the Government's responses (Section 9); and reflect on how A/NZ has managed the virus and the future challenges the country faces (Section 10).

Note that three key websites provide detailed information on COVID-19 in A/NZ (Ministry of Business Innovation & Employment, 2020; New Zealand Government, 2020*a*; Ministry of Health, 2021). The last includes all data on cases in A/NZ. Regular blogs from public health experts are available at Various (2020–2021). All dates refer to 2020, unless otherwise signified.

## 2. An overview and timeline of COVID-19 in Aotearoa New Zealand

A/NZ is a relatively small, island nation in the Western South Pacific, with a population of 5.1 million people. It has an Indigenous Māori population, who make up around 16.67% of the total population. A Treaty – Te Tiriti o Waitangi – signed between the Crown and Māori in 1840 plays a key role in Crown–Māori relations and policies. Pacific peoples make up 8.3% of the population (the largest groups being Samoan, Tongan and Cook Islands Māori), and there are close ties between A/NZ and Pacific nations. Asian peoples make up 15.7% of the population (the largest groups being Chinese, Indian and Filipino) (Stats NZ, 2020*a*).

As with the rest of the world, the A/NZ government (at the time, a Labour-Party-led Coalition government with the Green and New Zealand First parties) was watching closely as news of the virus began in late 2019/early 2020. The World Health Organization (WHO) declared the pandemic a global health emergency on 30 January 2020.

Early advice in A/NZ emphasised good hand hygiene (washing hands regularly, using sanitiser, not touching the face), cough etiquette (covering the mouth and nose) and people staying at home if they became sick, with advice to call a national Healthline over any health concerns. It also involved some early screening of arrivals at the border and encouraging those who had visited certain countries to self-isolate and register with Healthline.

A/NZ's response to the virus ramped up from February onwards. A timeline of key events is set out in Box 1.

Box 1. Timeline of key events for COVID-19 in Aotearoa/New Zealand

**Figure 1.**
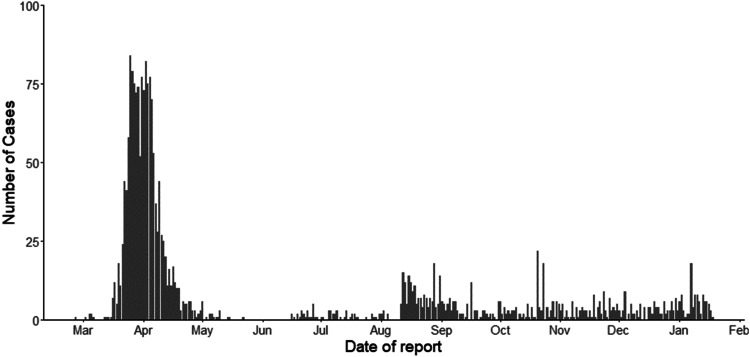
Daily confirmed and probable cases in A/NZ, as of 18 January 2021. *Source*: Ministry of Health (2021).

This box sets out key events surrounding COVID-19 in A/NZ, including case numbers at key points in time. Total case numbers are graphed in Figure 1.

3 February – Ban on the entry of foreigners from, or travelling through, China (the original source of the virus). This marked the beginning of the country's response to the virus, with travel and other restrictions intensifying over the next month, including requiring those entering the country to self-isolate for 14 days, and the banning of some gatherings and events.

21 February – First case of COVID-19, a person who had been to Italy.

28 February – First *reported* case of COVID-19, a person in their 60s recently returned from Iran.

5 March – First case of local transmission, an Auckland man infected by a family member recently returned from Iran.

19 March – Borders close; those returning to A/NZ to self-isolate for 14 days (at 39 cases).

21 March – Release of four-level Alert Level Framework (New Zealand Government, 2020*b*) (at 52 confirmed, 4 probable cases, a total of 56 cases); Country enters Level 2.

23 March – Country enters Level 3.

25 March – Country enters Level 4 (full lockdown).

29 March – First death (at 476 confirmed and 38 probable cases, a total of 514 cases).

9 April – All those arriving must go to a managed isolation facility for 14 days (at 992 confirmed and 247 cases, a total of 1239 cases).

27 April – Country returns to Level 3.

13 May – Country enters Level 2 (11 May, at 1147 confirmed and 350 probable cases, a total of 1497 cases).

8 June – Country enters Level 1.

11 August – 102 days since new cases in the community – all cases amongst those who had entered from overseas and all were in managed isolation (1220 confirmed and 350 probable cases, a total of 1570 cases and 22 deaths).

11 August – Four new cases in the community, in the country's largest city, Auckland. Auckland to Level 3, rest of country to Level 2.

21 September – Rest of country except for Auckland to Level 1.

8 October – Auckland to Level 1 (1508 confirmed and 356 probable cases, a total of 1863 cases and 25 deaths). This second outbreak, therefore, had led to a further 293 cases and 3 deaths.

Up to 12 November – Some cases in the community.

13 November – Those working in Auckland's central business district to work at home while further testing of community cases took place.

18 November – Last reported community case.

19 November – Aucklanders to wear masks on public transport and for all New Zealanders to wear masks on flights.

18 January 2021 – 1906 confirmed and 356 probable cases, for a total of 2262 cases, and 25 deaths.

## 3. Decision-making

The response to the pandemic has been led at the highest levels of government, involving the Prime Minister (Jacinda Ardern), the Minister of Finance (Grant Robertson), the Director-General of Health (Dr Ashely Bloomfield, a public health medicine specialist), alongside key Ministers whose roles have changed over time. Since November, a Minister for COVID-19 Response (Hon Chris Hipkins) has oversight. Cabinet signs off on key decisions.

An All-of-Government response group run out of the Department of the Prime Minister and Cabinet has co-ordinated the response since July 2020. A National Response Leadership Team therefore includes the Chief Executive of the Department of the Prime Minister and Cabinet; the Director-General of Health; Chief Executive of the National Emergency Management Agency; the Commissioner of Police; the Secretary of the Treasury; and the Deputy Chief Executive (COVID-19 Group) of the Department of the Prime Minister and Cabinet (Department of the Prime Minister and Cabinet, 2020) (Figure 1).

A raft of government agencies have been involved in the policy and implementation responses, including the Ministry of Health (public health and health services); the Treasury (finance, economy and budgets); the Ministry of Business, Innovation and Employment (business, employment and immigration actions); the Ministry for Social Development (welfare support); and the Police, Civil Defence and Emergency Management, and the Defence Force (enforcement, emergency management, and management of managed isolation and quarantine facilities). Key health service organisations have also been involved in the response, including the 20 District Health Boards (health services) and their associated Public Health Units (testing, tracing), laboratories (testing) and general practice, pharmacies and residential rest homes in ensuring the delivery of safe, essential health services.

The Ministry of Health has a COVID-19 Technical Advisory Group, consisting of epidemiologists and public health specialists, laboratory and general practice, Māori and Pacific health experts. The national ESR is contracted by the Ministry of Health to provide intelligence on COVID-19 in A/NZ, including research such as genomic testing and waste water testing (ESR, 2021).

## 4. International borders

On 3 February, A/NZ temporarily banned the entry of foreigners from, or travelling through, China; homecoming New Zealanders were exempt but required to self-isolate for 14 days. A chartered flight brought New Zealanders home from Wuhan on 4 February, with those arriving quarantined at a naval facility for 14 days. The travel ban was then extended to Iran on 28 February, and on 3 March, those from northern Italy and South Korea were having to self-isolate for 14 days. By 16 March, there had been eight cases, and all travellers (except those from a range of Pacific Islands, unless they were showing symptoms) were being asked to self-isolate. Restrictions were also put in place to prevent those with symptoms from travelling to neighbouring Pacific Islands. Cruise ships were also banned from entering the country.

On 20 March, A/NZ closed its borders to non-New Zealanders. By 25 March, all those returning home were to be screened, and those displaying symptoms were to be placed into managed isolation, while others were to self-isolate. Concerns over whether self-isolation was happening, meant that from 9 April all those arriving home were actively placed into managed isolation facilities (hotels) for 14 days.

Between mid-May and 11 August, the only cases were of people entering the country, all in managed isolation and tested on days 3 and 12 of their stays. On 11 August, however, four new cases were recorded in the community, a strain of the virus not seen in A/NZ before this time. It is thought that the virus entered via the border, but the source has never been found. From 13 August, those who tested positive for the virus were to be managed in quarantine facilities, with stronger security and health staff on site at all times.

A limited, one-way ‘bubble’ with Australia was introduced from 16 October. New Zealanders were able to travel (without going into managed isolation) to New South Wales, the Australian Capital Territory, and the Northern Territory, flying into Sydney, New South Wales. However, New Zealanders did travel internally within Australia, including Tasmania, Western Australia and the state of Victoria, which was still in lockdown. Victoria allowed New Zealanders to fly in from 9 November, and Queensland from 12 December. The travel arrangements have, however, altered depending on the spread of the virus in each Australian state. They operate only one-way, with those returning from Australia to A/NZ having to go into managed isolation for 14 days. It is expected that managed isolation may no longer be required by April 2021. A one-way ‘bubble’ from the Cook Islands is expected from 21 January 2021, although those travelling to the Cook Islands will still have to go into managed isolation for 14 days.

Most of those entering A/NZ are New Zealanders returning home, but there have been some exemptions (e.g., for humanitarian reasons, essential medical workers, shipping crews, and others with unique skills or needed for ‘time critical’ projects such as film crews who are then also supporting jobs in A/NZ). Between 26 March and 18 January, 99,438 people had been through the managed isolation process (Ministry of Business Innovation & Employment, 2020). Some returnees (e.g., those leaving A/NZ and returning within a short time) are now charged for isolation costs. Since 15 January 2021, all those on flights from the UK and USA have required negative COVID-19 tests completed at least 72 hours before boarding their planes; from 25 January 2021, these requirements will hold for all those arriving in A/NZ other than from Australia, Antarctica and most Pacific Islands.

There have been some issues with security at managed isolation hotels, with some people trying to leave or wandering the streets for several hours (Birkinshaw, 2020). In June, the Defence Force took over the oversight and management of such facilities (Murray, 2020). In late August, in order to further strengthen security, and following reports of staff falling asleep and data being leaked about returnees in managed isolation, the government increased the number of Defence Force staff working at such facilities, and, where security guards were being used, they would in future be employed by the government, with training and paid a living wage (Anthony, 2020).

Ensuring A/NZ's air and sea borders are tightly managed is the key means of keeping the country virus-free. The testing of staff at the border was identified by the media as inconsistent and slower to be implemented than the government expected (Mazey and Richardson, 2020), which has tightened requirements steadily over time. There are health checks on arrival for e.g., airline staff, and daily health checks for airport staff. Those working in quarantine facilities and transporting people to and from those facilities must now be tested every seven days; while those working in managed isolation facilities and related transport, and those in high-risk occupations at the ports, are to be tested every 14 days.

A/NZ is reaching limits on how many people can be in managed isolation facilities [around 5500 people (Ministry of Business Innovation & Employment, 2020)] and has been working with airlines to limit the numbers of people returning; this had become a particular issue as New Zealanders seek to return home for Christmas. The government has noted that the key limiting factor is the staffing of managed isolation facilities.

Some industries, however, are pointing to the effects of these limits on, for example, their ability to enable post-graduate students into the country and to fill pressing skill shortages (e.g., in health care, and in horticulture). On 27 November, it was announced that an existing scheme would allow 2000 people from the Pacific Islands to enter A/NZ to work in horticulture over the summer. On 14 January 2021, the government announced there would be places for 1000 students (already part-way through their courses) to enter managed isolation. This would assist in supporting the international education sector. Students would have to pay for their time in managed isolation.

## 5. Restrictions and alert levels

On 21 March (with 52 confirmed and four probable cases), the government requested New Zealanders who could, to work at home, and to limit travel. It asked those aged over 70 and with poorer health to stay home. It announced an Alert Level framework, with four levels, Level 1 the least restrictive (New Zealand Government, 2020*b*). A/NZ was put on Alert Level 2: with physical distancing (2 m), staying at home if possible, avoiding non-essential travel, and limits on indoor (100 people) and outdoor (500 people) gatherings.

Two days later, on 23 March (with a near doubling to 102 confirmed cases), A/NZ went to Alert Level 3 – with people to stay and work at home, and schools and non-essential services to close. There was 48 hours' notice of a move to Alert Level 4, to last four weeks.

On 25 March (with 205 confirmed and probable cases), A/NZ went into Alert Level 4 lockdown. This meant: staying at home with a small household bubble; staying local (e.g., for shopping, only at supermarkets and petrol stations, or for GP and pharmacy care); no public gatherings; closed public venues; closed education facilities; and essential items only (e.g., heaters, ovens, etc.) able to be purchased online from retail stores.

Some Māori iwi (tribes), in the north and east of the North Island, put in place their own road checkpoints to stop the spread of COVID-19 to their regions. They felt not enough was being done to protect their vulnerable communities, who have lower health status and face greater deprivation, than other populations. Māori had been hit hard by the influenza pandemic after the First World War and did not wish to suffer once more.

By 16 April, there had been 1084 confirmed and 317 probable cases (total: 1401) and nine deaths, but the number of new cases was below 30 each day.

On 20 April, the Prime Minister announced there would be a move to Alert Level 3 at 11.59pm, Monday 27 April. More businesses could open (e.g., construction) but with social distancing and hygiene requirements. Retail stores (including food outlets) could sell online only, with online payments, and contactless pick-up or delivery. Those who could work at home were asked to stay at home. Schools (years 1–10) and early childhood centres could open only for those students who needed to be there (e.g., whose caregivers were back at work). Alert Level 3 would be in place for 2 weeks.

On 7 May, the Prime Minister announced details on Alert Level 2 – with requests for those who could, to continue to work at home. Educational facilities would reopen. Travel around A/NZ was to be allowed.

On Monday 11 May, it was announced that A/NZ would move to Alert Level 2 from 11.59pm on Wednesday 13 May. Most businesses could then reopen, provided they had means of ensuring good physical distancing. Schools returned on Monday 18 May; bars could reopen from Thursday 21 May.

After 102 days with no cases in the community, four new cases, with an unknown source, were identified, in South Auckland. This led to Auckland being placed in lockdown Level 3 from midday 12 August, and the rest of the country in lockdown Level 2. Travel in and out of Auckland was severely restricted, via police roadblocks. On 30 August, Auckland stepped down to lockdown Level 2.5 (with lower gathering limits than the rest of the country). All but Auckland stepped down to Level 1 on 21 September; Auckland moved to lockdown Level 2 (easing gathering restrictions) on 23 September, then moved to lockdown Level 1 on 8 October. By that date, New Zealand had reached 1508 confirmed and 356 probable cases (a total of 1863) and 25 deaths.

Since then, in addition to regular cases from returnees to A/NZ, a few further community cases have been reported, all with links to the border (e.g., a marine engineer working on ships; a managed isolation worker employed where foreign shipping crews were isolating; a quarantine Defence Force worker in Auckland, where the virus spread to a number of other colleagues; a student, whose case was connected genomically to the Defence Force worker case; and an Air New Zealand crew member whose case is not linked genomically to other A/NZ cases) (Wilson *et al*., 2020). None of these cases resulted in changes to Alert Levels, although the 12 November case did result in the government asking all those working in Auckland's central business district to work at home, on 13 November, while further testing took place. As a result of these cases, the government introduced requirements (as of 19 November) for Aucklanders to wear masks on public transport and for all New Zealanders to wear masks on flights.

As of 18 January 2021, there had been no community cases since 18 November.

## 6. Cases, clusters and deaths

As of 18 January 2021, 50% of all cases to that date were imported, and a further 21% were related to those cases. Twenty-five per cent of cases were locally acquired with the source known, and 4% were locally acquired, with no overseas travel and no link to an identified case (see Table 1).

**Table 1.** Aotearoa New Zealand COVID-19 cases, 18 January 2021

Source of cases	Number	Percentage
Imported case (international travellers and crew)	1122	50
Import-related case (such as close contacts and border officials)	478	21
Locally acquired, source known	566	25
Locally acquired, unknown source	96	4
Total	2262	100

*Source*: Ministry of Health (2021).

A slightly higher proportion of cases has been amongst females (52.30%) and more cases have been amongst those 20–29 than amongst any other age group (23.83%). Europeans have made up 61.27% of cases, Asian 17.99%, Māori 8.62% and Pacific 8.13% of cases; the proportion of Asian peoples affected being slightly higher than their population proportion, and the proportion of Māori being quite a bit lower than their population proportion (see Table 2).

**Table 2.** Total cases, deaths and tests by age, gender and ethnicity, as of 18 January 2021

	Total cases	%	Total deaths	%	Total tests	%
Gender						
Female	1183	52.30	11	44.00	781,000	53.28
Male	1077	47.61	14	56.00	663,805	45.29
Unknown	2	0.09	0	0.00	20,953	1.43
Total	2262		25		1,465,758	
Age						
0–19	296	13.09	0	0.00	255,266	17.42
20–29	539	23.83	0	0.00	268,736	18.33
30–39	418	18.48	0	0.00	263,175	17.95
40–49	322	14.24	0	0.00	213,773	14.58
50–59	327	14.46	2	8.00	209,637	14.30
60–69	226	9.99	3	12.00	152,197	10.38
70+	134	5.92	20	80.00	102,974	7.03
Total	2262		25		1,465,758	
Ethnic group						
Maori	195	8.62	5	20.00	211,983	14.46
Pacific peoples	184	8.13	1	4.00	172,695	11.78
Asian	407	17.99	0	0.00	218,011	14.87
Middle Eastern/Latin American/African	80	3.54	1	4.00	NR	NR
European/other	1386	61.27	18	72.00	805,524	54.96
Unknown	10	0.44	0	0.00	57,545	3.93
Total	2262		25		1,465,758	

*Source*: (Ministry of Health, 2021).

Nationally, there have been 18 clusters of 10 or more cases of COVID-19, people who caught the virus off each other. These total to 830 cases (or 41% of the total). The largest cluster was of 179 people in the Auckland August cluster. One hundred and fifty-four cases were in five residential rest homes (Ministry of Health, 2021).

Of the 25 people who have very sadly died, two were aged under 60, three were aged 60–69, seven aged 70–79, eight aged 80–89 and five aged over 90. Seventy-two per cent of those who have died are of European ethnicity; 20% are Māori; 4% are Pacific; a higher death rate for Māori than their population proportion. Twelve deaths came from a single South Island rest home cluster (Ministry of Health, 2021).

## 7. Tracing and testing

Initially, testing at A/NZ's three laboratories could handle between 500 and 1000 tests per day; testing capacity has ramped up considerably since that time (Johnston, 2020) – for a few days in late August 2020, over 20,000 tests were being done each day.

Community testing initially focused on those with symptoms who had travelled overseas before becoming unwell and close contacts of those suspected or confirmed with COVID-19. Later, clinicians were advised to use clinical judgement as to whether someone should get tested; but, for a time at least, there appeared to be some confusion over the guidance and whether tests suggested by general practitioners would or would not be allowed at community testing stations, or whether they would be processed. Later still, in early April, testing widened to include all those with symptoms, and advice was that people could self-refer for a test. There has also been some targeted community testing (e.g., at supermarket carparks) to identify if the virus is in the asymptomatic population. Particularly during outbreaks, long queues formed for tests, and people were reminded that the priority would be for those with symptoms to be tested.

By 11 May, 194,191 tests had been completed. By 9 August, 494,481 tests had been completed. By 22 November, 1,230,091 tests had been completed, around 221 tests per 1000 people, including those in managed isolation and quarantine, with some people tested more than once (Ministry of Health, 2021).

A COVID-19 app was launched on 20 May, with the government asking people to download the app and scan Quick Response (QR) codes in community locations (e.g., cafes). From 19 August, businesses, and from 3 September, public transport operators, have been required to have a QR code. Use of the app has varied over time, and the government has frequently reminded New Zealanders to use it. For example, on one day in early September, there were 2.5 million scans from close to 1 million devices, but by early November, of the 2.3 million registered users of the government app, only 17% were using it, and by 16 January 2021, there were close to 480,000 scans from 270,000 active devices. A trial is underway for a COVID-19 card that would automatically (via Bluetooth) enable faster contact tracing should someone in the community contract the virus.

Contact tracing initially relied on capacity at 12 Public Health Units, which soon became stretched. In March, a National Close Contact Service, led by qualified nurses, was set up. An April rapid audit found that the system was overloaded at fewer than 100 cases per day, and tracing was taking an average of 2.3 days. It was recommended that additional resources be provided, and that information and monitoring flows improve to ensure the system is performing well (Verrall, 2020). The goal is for 80% of contacts of a person who has a positive COVID-19 test to be traced and quarantined within four days of exposure to the case, to prevent onward transmission (Ministry of Health, 2020*b*).

## 8. Capacity of the health system

Prior to COVID-19, it was recognised that A/NZ was not well placed to manage an epidemic or pandemic. Public health responsibilities are viewed as fragmented across agencies, and significantly under-resourced. A Global Health Security Index scored A/NZ 54/100 in a late 2019 report on pandemic preparedness, just above the high-income score of 51.9 (Nuclear Threat Institute and Johns Hopkins, 2019).

New funding for the health sector therefore came early for public health (17 March) (especially for contact tracing, doubling the resources available). There was also additional funding provided at this time for primary care (including community testing centres and assistance to increase telehealth services), Healthline and hospitals for intensive care capacity and equipment. The $500 million package also included (amongst other support) specific funding for Māori and Pacific responses and communities, for the purchase of personal protective equipment (PPE), for increasing pharmaceutical prices, for an elective services catch-up campaign and for a national vaccine strategy (RNZ, 2020*a*).

The government encouraged New Zealanders to get their flu vaccines early, concerned over a potential double-up of flu and COVID-19 swamping the health system. Requirements for many people to wait 20 minutes after their vaccination were relaxed to five minutes, with novel approaches taken to getting people vaccinated (e.g., in carparks).

There has been ongoing concern over access to appropriate PPE. General practices, midwives, those working in rest homes, personal care givers and nurses have all, at times, raised concerns around the adequacy of supply at practices around the country, and whether high-level P2/N95 masks should be worn instead of surgical masks (alongside gloves, gowns/aprons and eye protection). The Ministry of Health argued there was enough supply, but acknowledged there may be issues with distribution, which was later centralised. Concerns were still being raised as late as November, with nurses in managed isolation facilities arguing for the use of N95 masks as opposed to surgical masks. On 26 November, it was announced that staff doing daily health checks, transporting cases or working in managed isolation or quarantine within 2 metres of those infected or potentially infected should wear N95 or P2 masks. Such staff are to be trained in the proper use of the masks.

A/NZ identified there were 153 ICU beds available, with the potential to repurpose to over 560 beds. There was access to 520 ventilators. It was felt there would be sufficient staffing for these facilities, especially once hospitals began to postpone elective services and train the released staff to care for people on ventilation. However, the capacity of the health system was never really tested as the number of cases has remained relatively low throughout. One hundred and twenty-two people were hospitalised with COVID-19, 17 spent time in ICUs (Ministry of Health, 2021).

Throughout the pandemic, thousands of extra health professionals came back into the workforce and were given additional training to support e.g., the delivery of health services, testing and contact tracing. However, especially during lockdown, patient numbers fell dramatically in primary care (Ministry of Health, 2020*c*), raising concerns over the viability of GPs. Many primary care and outpatient services turned to use telehealth services. It has been reported that most cancer services continued appropriately, but that diagnostic services significantly reduced (Te Aho o Te Kahu, 2020). Elective services were also affected during the lockdowns (Ministry of Health, 2020*c*). As a result, there is a need for a significant increase in health services delivery to catch up, and additional funding ($282.5 million over 3 years, out of a total health budget of around $20 billion) has been provided to the health sector to support that.

## 9. The economic impact and response

The A/NZ economy has been hit hard by COVID-19. International tourism makes up around 5.8% of GDP, and tourism and hospitality businesses have lost significant business with no tourists and through lockdowns. Domestic tourism is being encouraged but cannot make up for the 3.6 million tourists that visited in the year to March 2020, nor their spending (which is higher than for domestic visitors). The international education sector (contributing around $5 billion per annum in 2018) has also been hit hard by COVID-19. Exports have held up well, however, during the year, while imports have plummeted, improving A/NZ's overall balance of trade. The trade weighted index – measuring the value of the currency compared to the country's main trading partners – fell during the lockdowns in A/NZ in 2020, but has since recovered.

In the March–June quarter, GDP fell by a record −12.2% compared with the same quarter the previous year, although this was lower than originally forecast (The Treasury, 2020*a*). This is just above the OCED average (e.g., Australia's was −6.3%), but lower than the UK's fall of 21.7% (Stats NZ, 2020*b*). The economy has also bounced back more quickly than anticipated (with The Treasury predicting a 10.5% rebound for July–September). The Treasury recorded a fall in annual average GDP of −2.1% in the year to the end of June 2020 and is now predicting a growth of 1.5% in the year to the end of June 2021.

Unemployment is predicted to peak to 6.9% by the end of 2021, up from 4% at the beginning of 2020. The impact, however, of unemployment and an increase in the number of people on government benefits is expected to adversely affect young people, women, Māori and Pacific peoples, and those living in Auckland (The Treasury, 2020*a*).

Net core crown debt as a percentage of GDP is expected to increase from 19% to the end of June 2019 and 26.4% in 2020, to 39.7% in 2021 and to a high of 52.6% in 2023 (The Treasury, 2020*a*).

A COVID Response and Recovery fund of $NZ 50 billion (around 16% of A/NZ's annual GDP) has been established to combat the effects of COVID-19 (The Treasury, 2020*b*). Key programmes have included a wage subsidy for businesses to continue to pay employees and support sick leave and self-isolation at home, additional income support including a higher benefit for those losing their jobs as a result of COVID-19, changes to business taxes, a business loan support scheme, mortgage repayment holidays for homeowners and small and medium businesses, and an aviation support package, to protect supply chains in and out of A/NZ, including a low interest loan to Air New Zealand, partly owned by the government. The May budget included new funding to support key sectors (e.g., primary industries, arts and culture, etc.). The emphasis is on providing government funding to achieve key infrastructure goals, such as building many new houses to respond to a major housing shortage, improving roading and rail, supporting innovative industries, investing in local infrastructure such as water supplies and distribution, and improving the environment through planting, conservation and improvements to waterways.

## 10. Reflections on the Aotearoa New Zealand response

The A/NZ government went ‘hard’ and ‘early’ in its response to COVID-19. The approach has been highly successful, with A/NZ in the bottom 10 countries by deaths per capita (Johns Hopkins, 2020), one of the lowest rates of cases per capita internationally (World Health Organization, 2020*a*), and ranked top in a recent Bloomberg resilience ranking (which takes into account 10 key metrics, including growth in virus cases, the overall mortality rate, testing capabilities, vaccine supply agreement, capacity of the health care system, the impact of restrictions on the economy and citizens' freedom of movement) (Bloomberg, 2020).

Several key factors have assisted A/NZ in keeping the virus at bay. The country is isolated geographically, and, as an island nation, has been able to close its borders with relative ease. The country has a low population density, and nowhere near as crowded public transport systems as many other countries, making physical distancing easier.

There is no doubt, however, that the government's response has also contributed significantly to the country's success. It acted quickly and resolvedly, locking the country down relatively early as the virus began to spread in March, and not hesitating to lock the largest region (Auckland) down again in August when the virus was once again spreading in the community.

The response has been strongly informed by science, with advisors regularly reviewing the situation and evidence locally and internationally, and the government making decisions on the basis of the situation each day. The response has been a learning one, with various policies tweaked over time in response to concerns and issues, including those raised by the media (e.g., relating to security at managed isolation hotels).

The government's communication has been exceptionally strong, with daily televised updates, usually from the Prime Minister and Director-General of Health (a public health medicine specialist). The government has emphasised that ‘we are all in this together’ and the need for ‘the team of five million’ to recognise the threats posed by the virus, to follow the rules, and to exhibit ‘kindness’. There have been regular commentaries by a range of experts, and regular reporting around international experiences, putting A/NZ experiences into an international context. This high-level leadership has recently been bolstered by the appointment of a specific COVID-19 Response Minister.

A clear framework for the response – the Alert Level Framework – was released early in the pandemic, setting out what the implications of each Alert Level would be. It did take time, however, for clarity over some key points, for example, over exactly which businesses qualified as essential services or what some of the measures might mean at different levels e.g., in schools, but in most cases, it took only a few days for the rules or arrangements to become clearer. The signalling of when key decisions on Alert levels would be made has also assisted in the response.

Most New Zealanders have supported the approach taken by the government and have adhered to advice and the Alert Level guidelines. A Horizon poll in late August found 79% of people trusted the A/NZ response, albeit this had fallen from 91% in April and 82% in July, affected by the second Auckland lockdown (Horizon Poll, 2020). In late 2020, the three-yearly national election was held, delayed as a result of COVID-19. With 82% of those eligible voting, the Labour Party was elected to power with a landslide victory, winning 50% of the overall vote, and allowing the Labour Party to govern on its own, something which has not happened in A/NZ since the introduction of a Mixed-Member Proportional voting system in 1996. This demonstrates how successful the Party – and its leader, Jacinda Ardern – has been in managing COVID-19.

Although the response was ‘early’ and ‘hard’, the government has taken a more conservative approach than some experts would have liked.

Much of the debate has focused on the country's responses at the border. It has been suggested, for example, that a slightly earlier and stronger approach at the border, such as earlier testing of all arrivals and earlier use of self-isolation for those returning from high-risk countries, may have limited the impact of the virus even more in A/NZ, pointing to Taiwan's success in managing COVID-19 through such early actions (Summers *et al*., 2020). Indeed, the Ministry of Health initially supported closing A/NZ's borders all together, but that approach was rejected as it would have left New Zealanders stranded overseas (RNZ, 2020*b*). More recently, a risk-based, traffic-light approach has been suggested; this would take into account the levels of virus spread within other countries and place more restrictions on those coming from higher-risk countries (e.g., pre-flight isolation to go with the now introduced negative pre-flight tests) [Wilson and Baker, 2020 (Nov 4)].

Another area of contention has been around the wearing of masks. Some experts had been suggesting for months that people should be encouraged, or mandated, to wear masks in higher-risk situations (e.g., on public transport, at indoor venues) (Kvalsvig *et al*., 2020), but the policy was not introduced until August, following updates of international evidence and a change of views at the WHO (World Health Organization, 2020*a*, 2020*b*) and Ministry of Health (Hancock, 2020).

Some have criticised the implementation of key features of COVID-19 policy; for example, the security lapses that allowed people out of managed isolation (Mazey and Richardson, 2020); the use and distribution of PPE, which remains of concern [Kenny, 2021 (16 Jan)]; the slow introduction of regular tests for border workers [Daalder, 2020 (21 Aug)]; and the fact that many deaths occurred in residential rest homes. It has been noted, however, that the government has responded swiftly to a number of issues, drawing on expertise to assess and reorganise key processes in order to keep people safe (Mazey and Richardson, 2020).

Finally, a key criticism is that A/NZ was simply not prepared for such a pandemic. The country's readiness had, in late 2019, been noted to be fairly poor, and the country relied on an influenza responsiveness plan as its starting point. And although the government went ‘hard’ and ‘early’ with respect to lockdowns, policy development and implementation has developed incrementally over the past year. The government is promising a new Public Health Agency as part of major reforms to the health sector, and preparedness for future pandemics will no doubt be a key part of its work. Not every possibility can be planned for, however, and resilient, flexible and thoughtful public policy institutions will be needed to handle the unexpected (Mazey and Richardson, 2020).

Overall, as of 19 January 2021, A/NZ can claim to have succeeded in its goal to eliminate the virus in the A/NZ community. This has been particularly impressive given the speed with which the pandemic worsened, with new and more contagious strains, late in 2020. Ongoing vigilance is needed to keep the virus out of the community, until a vaccination programme can begin, now scheduled from around April 2021. Planning is underway for the roll out of vaccines, first to border workers and health staff, followed by those more at risk, and the remainder of the population in the second half of 2021. The government has announced that there will be no charge for vaccinations.

There are concerns being raised within A/NZ (Heatley, 2020; Lally, 2020) and internationally (Goldblatt *et al*., 2020; Ilzetzki and Moll, 2020) over the medium- and longer-term economic and social implications associated with COVID-19 and various governments' responses to the virus, including around the costs and benefits of policy decisions and the potential impact on inequities. In A/NZ, there have been significant economic and social costs from border closures and lockdowns (with jobs being lost through a lack of international tourism, international students and in the hospitality sector). But it is still too early to judge how the A/NZ economy will perform overall, and in comparison with other countries that took different decisions in managing COVID-19. Certainly, the economic cost over the next few years will be significant, but it is also seen as offering opportunities to fill key infrastructure gaps (see above, Section 9) and to support and work with the private sector to reorient the economy to delivering higher-value products, in more environmentally friendly ways (Greenaway-McGrevy *et al*., 2020).

As A/NZ enters 2021, New Zealanders can be proud of what has been achieved in managing the virus. Once the crisis is over, and the vaccine has hopefully been successfully administered to the population, attention will turn to when and how to open the border, strengthening the economy by allowing international tourists and students back in, while also keeping the virus at bay. Particular attention will need to be paid to supporting those affected economically by the virus, and in ways that support improvements in equity.

It is also to be hoped that a thorough review of the country's response will be undertaken, and steps taken to ensure that the key lessons learned and policies and processes put in place can be quickly re-introduced should a future pandemic strike.
